# Protective Effects of GIC and S-PRG Filler Restoratives on Demineralization of Bovine Enamel in Lactic Acid Solution

**DOI:** 10.3390/ma13092140

**Published:** 2020-05-06

**Authors:** Naoyuki Kaga, Futami Nagano-Takebe, Takashi Nezu, Takashi Matsuura, Kazuhiko Endo, Masayuki Kaga

**Affiliations:** 1 Section of Fixed Prosthodontics, Department of Oral Rehabilitation, Fukuoka Dental College, Fukuoka 814-0193, Japan; matsuurt@college.fdcnet.ac.jp; 2 Oral Medicine Research Center, Fukuoka Dental College, Fukuoka 814-0193, Japan; 3 Division of Biomaterials and Bioengineering, Department of Oral Rehabilitation, School of Dentistry, Health Sciences University of Hokkaido, 1757 Kanazawa, Ishikari-Tobetsu, Hokkaido 061-0293, Japan; nagano23@hoku-iryo-u.ac.jp (F.N.-T.); tnezu@hoku-iryo-u.ac.jp (T.N.); endo@hoku-iryo-u.ac.jp (K.E.); sp8c5fx9@salsa.ocn.ne.jp (M.K.)

**Keywords:** pH neutralization, ion release, enamel demineralization, glass ionomer cement, surface pre-reacted glass ionomer filler

## Abstract

This study was aimed at investigating the protective effects of glass ionomer cement (GIC) and surface pre-reacted glass ionomer (S-PRG) fillers used as dental restorative materials on demineralization of bovine enamel. GlasIonomer FX ULTRA (FXU), Fuji IX GP Extra (FIXE), CAREDYNE RESTORE (CDR) were used as GICs. PRG Barrier Coat (BC) was used as the S-PRG filler. They were incubated in a lactic acid solution (pH = 4.0) for six days at a temperature of 37 °C. The mineral was etched from the enamel surface, and a large number of Ca and P ions were detected in solution. The Al, F, Na, Sr, and Sr ions were released in GICs and S-RPG fillers. The Zn ion was released only in CDR and the B ion was released only in BC. The presence of apparent enamel prism peripheries was observed after six days of treatment for the group containing only enamel blocks. pH values for the FXU, FIXE, CDR, BC, and enamel block groups after six days were 6.5, 6.6, 6.7, 5.9, and 5.1, respectively. Therefore, the observed pH neutralization effect suppressed progression of caries due to the release of several ions from the restoratives.

## 1. Introduction

Various bioactive materials have been explored for possible uses in dental applications, owing to the recent developments of novel dental materials and procedures. In particular, the effects of these bioactive materials on the stability and longevity of dental restorations as well as on the oral pathogens inducing dental caries were considered [1]. Dental caries, one of the most widely spread preventable chronic diseases [2], is caused by the organic acids produced as byproducts of the metabolism of fermentable carbohydrates by oral bacteria, which disturb the calcium phosphate balance of the enamel and dentine in a low-pH intraoral environment [3]. Active caries development generally occurs at pH < 4.9 under the action of lactic acid. However, this process stops when the pH of the medium increases to 5.7 [4]. Previously, chemical modifications of the restoration material surface that might prevent bacteria adherence and suppress plaque and biofilm formation have been proposed. For this purpose, novel biomaterials that enable slow release of chemicals or ions lethal to bacterial cells must be developed [5]. The concept of “minimum intervention dentistry”, formulated in 2000, emphasized that caries treatments should aim at both preserving the tooth structure through noninvasive procedures and detecting suspicious early lesions [6]. The currently-adopted diagnosis and treatment policy primarily focuses on self-healing through remineralization, which is based on the application of bioactive materials before employing a restorative approach that requires the removal of tooth tissue [7].

The main purpose of developing bioactive and antibacterial materials is to prevent the damage incurred by hard tissues. Glass ionomer cements (GICs) have been widely used for the conservative treatment of enamel and dentin because of their beneficial properties, such as strong chemical bonding to the tooth substrate and their ability to release fluoride ions [8,9]. The commercially available GICs typically comprise aluminosilicate-based glass and copolymers of polyacrylic acid and itaconic acid. The major components of the glass used to formulate GIC was composed of SiO_2_, Al_2_O_3_, AlF_3_, CaF_2_, or SrO_2_ [10]. Furthermore, GICs produce significant amounts of aluminum, calcium, fluoride, sodium, strontium, and silicate ions in neutral or acidic solutions over time and can effectively neutralize lactic acid solutions [11,12]. To shorten, the notation of Al, Ca, F, Na, Sr, and Si ions were used hereafter. The important role of ion release is preventing demineralization of the areas adjacent to the tooth surface and suppressing the formation of initial caries. GICs exhibited an inhibiting effect induced by the secondary caries at the restoration margins in the enamel and dentin in vitro [13].

Surface pre-reacted glass ionomer (S-PRG) fillers were introduced as bioactive materials that were able to retain their basic properties by forming a stable glass ionomer phase on the treated surface through the acid–base reaction between the fluoro-boro-alumino-silicate glass and polyacrylic acid [14]. S-PRG fillers can induce release of the ions, namely: Al, F, Na Sr, Si ions, as well as borate ion (B ion). These fillers represent a novel class of particle materials that can be incorporated into resin matrices to produce a strong bioactive effect [15]. The results of the in vitro studies demonstrated the effectiveness of such materials in inhibiting the demineralization of the tooth substrate [16,17,18] and preventing the initiation of caries. In addition, an endodontic root canal sealer containing an S-PRG filler (i.e., S-PRG sealer) exhibited good antibacterial and anti-inflammatory properties. Finally, the implantation of S-PRG sealer into the subcutaneous tissues of rats noticeably decreased their inflammatory responses [19]

The primary objective of this study was to examine the inhibitory effects of four products (three well-known GICs and one S-PRG containing coating material) on enamel demineralization to evaluate their anticaries properties. The release ions from GICs and S-RPG fillers and the acid buffering capacity were examined, and the surfaces of bovine enamels were observed by scanning electron microscopy (SEM). The null hypothesis was that the GIC and S-PRG fillers would not inhibit the demineralization of bovine enamel specimens that were incubated in a lactic acid solution with pH = 4.0.

## 2. Materials and Methods

### 2.1. Preparation of Bovine Enamel Blocks

**Figure 1:**
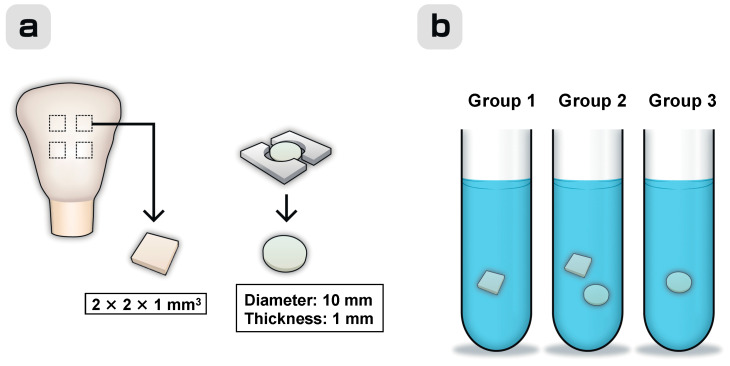
Schematic of the experimental procedure. (**a**) Preparation of enamel blocks from bovine crowns and restorative discs. (**b**) Test specimens classified into the following groups: enamel blocks only (Group 1), restorative discs with enamel blocks (Group 2), and restorative discs without enamel blocks (Group 3). All specimens were immersed in 5 mL of the lactic acid solution with pH = 4.0.

Figure 1 shows the schematic of the utilized experimental procedure. The flattened enamel surface on the buccal aspect of the crown part of a bovine upper central incisor (Yokohama Meat Corporation, Yokohama, Japan) was exposed longitudinally with a low-speed diamond saw (Isomet, Buehler, Lake Bluff, IL, USA) using water as a coolant. The exposed enamel surface was polished with waterproof 600-, 1200-, and 2000-grit silicon carbide abrasive papers (Sankyo Rikagaku Co., Ltd., Saitama, Japan) under running water to increase its smoothness and then cut into slabs with thicknesses of approximately 1.0 mm. The dentin side was painted with nail varnish, and the slabs were cut into enamel blocks with dimensions of approximately 2 mm × 2 mm using a diamond saw, which were subsequently cleaned for 20 s in distilled water with an ultrasonic cleaner. Four enamel blocks were obtained from each bovine crown (Figure 1a).

### 2.2. Preparation of Restorative Discs and Arrangement of Specimens into Test Groups

The test specimens were classified into the following three groups (Figure 1b): enamel blocks only (Group 1), restorative discs with enamel blocks (Group 2), and restorative discs without enamel blocks (Group 3) (*n* = 6 for each group). The formulations and chemical compositions of all the commercially available materials used in this study are listed in Table 1 (this information was provided by the respective manufacturers). Three high-viscosity GICs were mixed as per their manufacturer’s instructions at a temperature of 23 ± 2 °C. The freshly mixed cement was placed in a stainless-steel split mold to form disc-shaped specimens with internal diameters of 10 mm and thicknesses of 1 mm. The cement was covered with a polyethylene sheet and compressed using a metal plate. The resulting mold was stored in an incubator for 1 h at 37.0 °C, after which the polyethylene sheet was removed. The obtained disc-shaped GIC specimens were stored in an environment with a 100% relative humidity and temperature of 37 °C for 24 h before immersion in 5 mL of the lactic acid solution at 37 °C for different periods. To produce discs containing S-PRG coating material (PRG Barrier Coat), four ampoules of the base and active components were mixed using a microbrush. The resultant mixture was placed in a stainless-steel split mold and illuminated with a light-curing unit (BlueShot, Shofu Inc., Kyoto, Japan) for 60 s from both sides. Acrylic resin discs of the same size were used as controls.

### 2.3. pH Measurements, Enamel Demineralization, and SEM Observations

**Figure 2:**
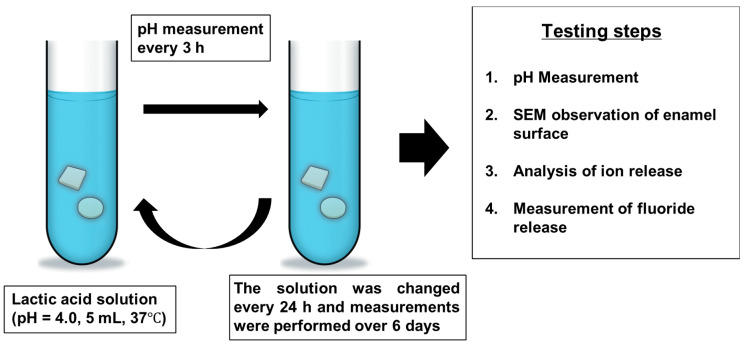
Description of the experimental methodology utilized for comparing the effects of glass ionomer cement (GIC) and surface pre-reacted glass ionomer (S-PRG) fillers on the demineralization of bovine enamel during incubation in the lactic acid solution with pH = 4.0 for six days at 37 °C.

The utilized experimental methodology is described in Figure 2. DL-lactic acid (Wako Pure Chemical Industries, Ltd., Osaka, Japan) was diluted with distilled water to 0.2 mM in order to adjust its pH to 4.0. Next, 5 mL of the resulting solution was pipetted into 50-mL glass conical tubes (Corning, Brooklyn, NY, USA). Specimens from each group were immersed in these tubes and incubated at 37 °C. The pH of each solution was measured every 3 h within a 24-h period. Afterwards, the specimens were transferred to tubes containing fresh lactic acid solutions and incubated for another 24 h at 37 °C. This process was repeated over a period of six days. To conduct pH measurements, a pH electrode (Orion 8102BNUWP, Thermo Fisher Scientific, Waltham, MA, USA) connected to a pH/ion meter (Orion 2115010 Dual Star pH/ion meter, Thermo Fisher Scientific, Waltham, MA, USA) was placed at the center of the conical tube. After six days of incubation, the enamel blocks were removed from the tubes and examined by SEM. For this purpose, they were rinsed with distilled water, dehydrated in a graded series of ethanol solutions (60%–100%), sputter-coated with Au, and observed under a scanning electron microscope (JEOL JCM-600 Plus NeoScope, JEOL, Tokyo, Japan) at an accelerating voltage of 5 kV.

### 2.4. Fluoride Ion Release Measurements

After the pH measurements (performed every 24 h), the amount of fluoride ions produced by various specimens was determined using a fluoride ion-selective electrode (Orion 9609 BNWP, Thermo Fisher Scientific, Waltham, MA, USA) that was attached to an ion analyzer (Orion 2115010 Dual Star pH/ion meter, Thermo Fisher Scientific, Waltham, MA, USA). In this measurement, 1 mL of each solution was pipetted into the wells of a 24-well dish. Subsequently, 10 vol.% of TISAB III buffer (Thermo Fisher Scientific, Waltham, MA, USA) was added to the wells to decomplex the fluoride complexes for ensuring that the F^–^ concentration can be determined correctly. The solution mixture in the wells was stirred gently for 5 min and then subjected to the fluoride ion measurements.

### 2.5. Analysis of Released Ions

After the completion of the pH and fluoride ion measurements, the solutions collected after 1 and six days of incubation were subjected to an ion analysis procedure. Al, B, Ca, Na, P, Si, Sr, and Zn ion release profiles were obtained by inductively coupled plasma-optical emission spectroscopy (ICP–OES) using an Optima 5300 DV system (Perkin-Elmer, Waltham, MA, USA). ICP–OES calibration standards were prepared from their corresponding stock solutions on a gravimetric basis. Three target calibration standards were obtained for each ion type, while deionized water and the lactic acid solution (pH = 4.0) were used as controls.

### 2.6. Statistical Analysis

All data were presented as mean ± standard deviation (*n* = 6). The obtained data sets (pH levels, Ca and P ion release profiles, and fluoride ion release measurements) were compared by performing the Kruskal–Wallis test, which was coupled with the Dunn’s multiple comparison post hoc test; herein, values with *p* < 0.05 were considered statistically significant. The differences between the Al, B, Na, Si, Sr, and Zn release profiles recorded after one and six days of incubation were analyzed using the Mann–Whitney test; values with *p* < 0.05 were considered statistically significant here as well. All statistical analyses were performed using GraphPad Prism software, version 8.1.2 (GraphPad Software, Inc., La Jolla, CA, USA).

## 3. Results

### 3.1. Release Profiles of Ca and P Ions

**Figure 3:**
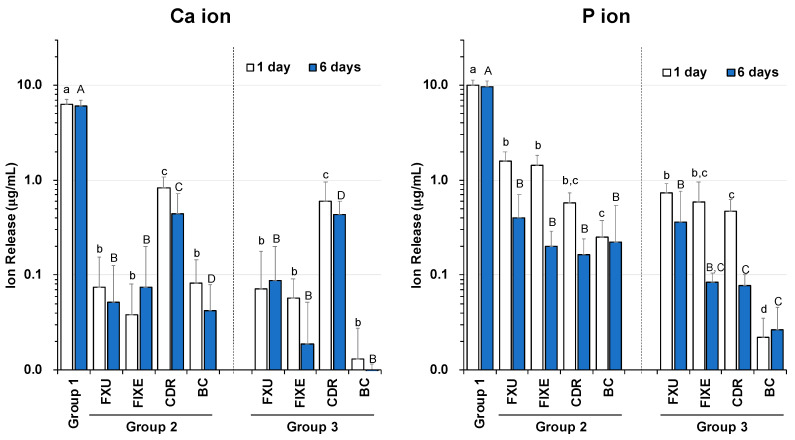
Ca and P ion release profiles. The solutions containing enamel blocks (Group 1) exhibit high Ca and P concentrations. The solutions containing CDR discs with and without enamel blocks (Groups 2 and 3, respectively) have higher Ca concentrations as compared with those of the solutions containing the other discs. Statistical differences were assessed by performing Kruskal–Wallis test with Dunn’s multiple comparison post hoc test (after one and six days of incubation). Values with *p* < 0.05 were considered statistically significant. The bars with same letters are not significantly different (*p* > 0.05). FXU: GlasIonomer FX ULTRA, FIXE: Fuji IX GP Extra, CDR: CAREDYNE RESTORE, BC: PRG Barrier Coat.

Figure 3 shows the obtained Ca and P ion release profiles. The highest amounts of released Ca ions were observed for the solutions containing only enamel blocks (Group 1); they were equal to 6.3 µg/mL after one day and 6.1 µg/mL after six days of incubation (*p* < 0.01). The amounts of Ca ions released from CDR were higher than those released from FXU, FIXE, and BC (Groups 2 and 3) (*p* < 0.05). For instance, their magnitudes determined for the solutions containing CDR discs and enamel blocks (Group 2) were equal to 0.8 µg/mL after one day and 0.4 µg/mL after six days. Meanwhile, for the solutions containing CDR discs without enamel blocks (Group 3), the concentration of released Ca ions was 0.6 µg/mL after one day and 0.4 µg/mL after six days of incubation. For the solutions containing FXU, FIXE, and BC disks with and without enamel blocks, the amounts of released Ca ions were below 0.1 µg/mL after both one and six days of treatment. Thus, the contents of Ca ions released by the enamel blocks during the incubation with the restorative materials (Group 2) were very low as compared with the values obtained for Group 1 (*p* < 0.05). Their magnitudes determined for Group 3 were also very small.

The highest amounts of detected P ions were obtained for the solutions containing only enamel block (Group 1); their values were equal to 10.5 µg/mL after one day and 10.2 µg/mL after six days (*p* < 0.01). The discs in Group 2 released ten times more P ions as compared to those released in Group 1; their contents after one day of incubation were 1.6 µg/mL for FXU, 1.4 µg/mL for FIXE, 0.6 µg/mL for CDR, and 0.3 µg/mL for BC (*p* < 0.05) (all these values decreased after six days of treatment). Thus, the amount of P ions released by BC (Group 3) after one day of incubation was the lowest one. The relatively small P contents obtained for Group 2 can be attributed to the dissolution of various components of the demineralized enamel. The trends observed for the amounts of P ions released in Groups 2 and 3 were very similar; however, the concentrations of P ions released in Group 3 were smaller than those released in Group 2 by more than a factor of 2. Finally, the average amount of released P ions noticeably decreased over the period between days one and six.

### 3.2. Release Profiles of Al, B, Na, Si, Sr, and Zn Ions

**Figure 4:**
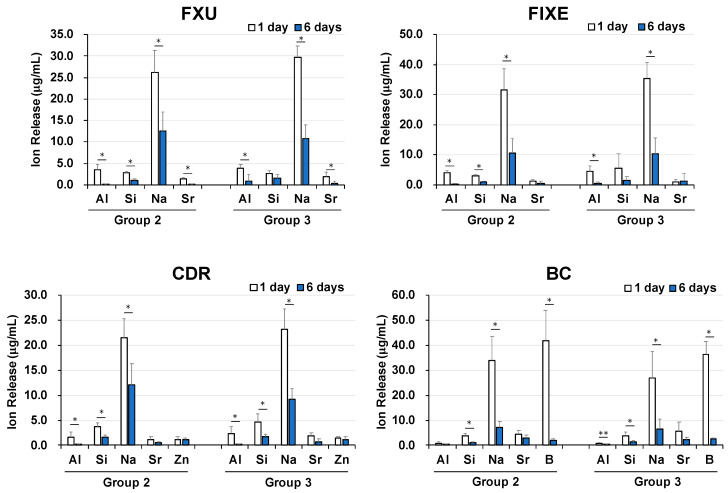
Al, B, Na, Si, Sr, and Zn ion release profiles. Concentrations of Al, B, F, Na, Si, and Sr ions in the solutions containing FXU, FIXE, CDR, and BC discs with and without enamel blocks (Groups 2 and 3, respectively) determined after one and six days of incubation. The differences in values obtained after one and six days were analyzed using the Mann–Whitney test. * *p* < 0.01, ** *p* < 0.05.

Figure 4 shows the Al, B, Na, Si, Sr, and Zn ion release profiles obtained for the solutions containing FXU, FIXE, CDR, and BC disks with and without enamel blocks after one and six days of incubation. The Al, Si, Na, and Sr ion release profiles of all the tested materials and their changes observed with increasing exposure time look very similar. The amounts of ions released by FXU, FIXE, and CDR after both one day and six days of incubation can be arranged in the order of Na > Al > Si > Sr. More importantly, the concentrations of Zn ions released in the CDR solutions (Group 2) determined clear release and were equal to 1.4 µg/mL after one day and 1.1 µg/mL after six days. BC exhibited the highest Na and B release rates (followed by those of Si and Sr ions), which decreased significantly between days one and six (*p* < 0.01). Finally, the number of released Al ions was very small, and their concentration decreased significantly over the period from day one to day six (*p* < 0.05).

### 3.3. Fluoride Ion Release

**Figure 5:**
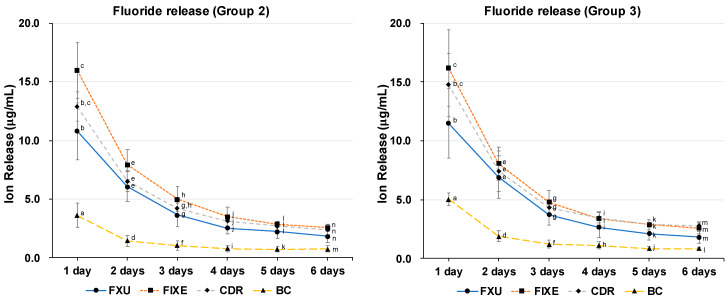
Fluoride ion release profiles. The concentrations of released fluoride ions decreased over time for all GICs in Groups 2 and 3. The fluoride content in the BC solution was significantly lower than those in the GIC solutions (*p* < 0.05). Statistical differences between various materials were assessed by performing the Kruskal–Wallis test with the Dunn’s multiple comparison post hoc test. Values with *p* < 0.05 were considered statistically significant. The bars with same letters are not significantly different (*p* > 0.05).

Figure 5 shows the fluoride release profiles obtained for specimen Groups 2 and 3. The highest amount of fluoride ions released after one day was observed for FIXE (*p* < 0.05) followed by CDR and FXU. However, their magnitudes sharply decreased after three days of incubation and then remained constant till day six. Furthermore, the concentrations of fluoride ions released in the FXU and CDR solutions were not significantly different over the entire measurement period (*p* > 0.05). The fluoride content released in the BC solution was approximately half of those released in the GIC solutions regardless of the measurement time, and their differences were significant (*p* < 0.05). Finally, the concentrations of fluoride ions released in Groups 2 and 3 exhibited similar trends.

### 3.4. pH Variations

**Figure 6:**
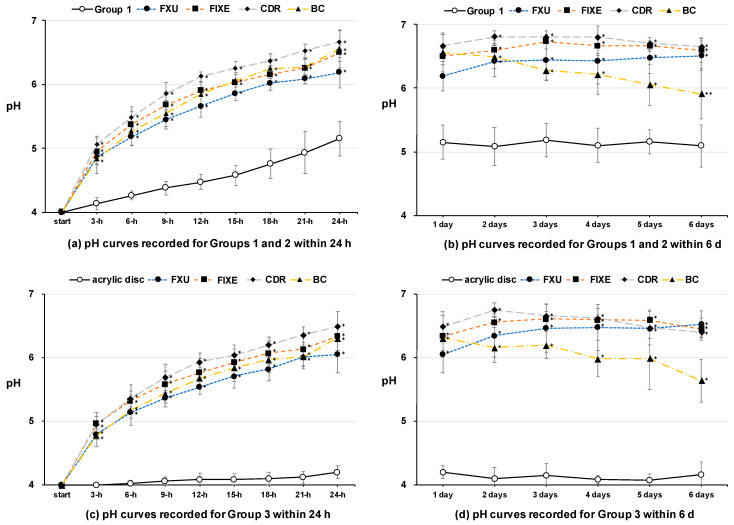
Variations of the pH values observed for different specimen groups. pH curves recorded for Groups 1 and 2 after incubation for (**a**) up to 24 h and (**b**) over a period from 24 h to six days. pH curves recorded for Group 3 after incubation for (**c**) up to 24 h and (**d**) over a period from 24 h to six days. Data were statistically analyzed by performing the Kruskal–Wallis test with the Dunn’s multiple comparison post hoc test ((**a**,**b**): control = Group 1; (**c**,**d**): control = acrylic disc; *n* = 6, mean ± SD). * *p* < 0.01, ** *p* < 0.05.

Figure 6 shows the results of the conducted pH measurements. For the solutions containing only enamel blocks (Group 1), the initial pH increased slowly from 4.0 to 4.1 after three hours and ultimately to 5.2 after 24 h of treatment. For the solutions containing GIC and S-PRG discs as well as enamel blocks (Group 2), the pH steadily increased to more than 4.9 after three hours, then to 5.2 after six hours, and finally to 5.7 after 12 h of incubation (Figure 6a). Between days one and six of measurements, the pH of Group 1 was approximately 5.1, while that of Group 2 was 5.9–6.8 (Figure 6b). Figure 6c shows the variations in pH of the solutions that contained only discs of the restorative materials during the first 24 h; whereas, Figure 6d displays the changes in pH between 24 h and six days. These graphs indicate that the pH variations of Group 2 were much larger than those of Group 1 at all measurement times (*p* < 0.01). In addition, the pH change for Group 3 was more significant than that for the control (acrylic disc) at all measurement times (*p* < 0.01). The S-PRG fillers constituting the BC were revealed to be inferior to the GIC restoratives with regard to the pH buffering capacity. Finally, the variations in the pH values of Groups 2 and 3 exhibited the same trends at all measurement times.

### 3.5. SEM Observations

**Figure 7:**
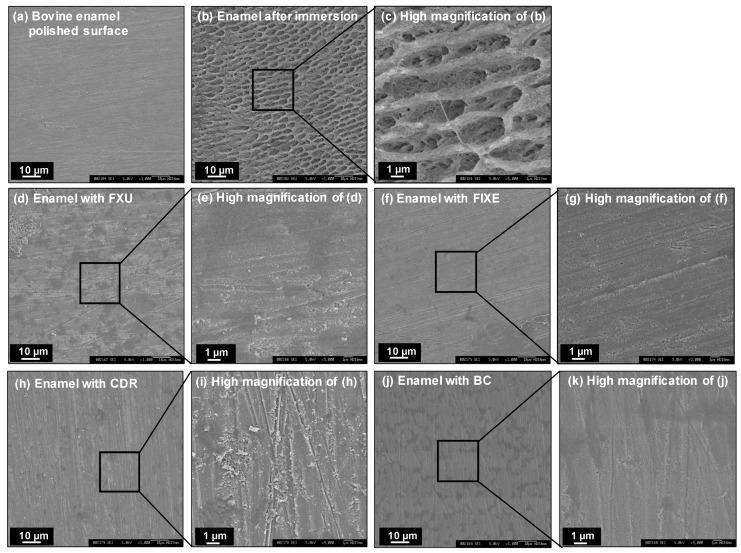
Representative scanning electron microscopy (SEM) images of the enamel surfaces obtained after six days of incubation. (**a**) Smooth and polished surface of the bovine enamel block. (**b**) Demineralized enamel after the sixth-day incubation in the lactic acid solution with pH = 4.0 (Group 1). (**c**) High-magnification image of the sample surface depicted in panel (b) (×5000). Non-damaged surfaces of the enamel blocks incubated with (**d**) FXU, (**f**) FIXE, (**h**) CDR, and (**j**) BC disks (Group 2). (**e**,**g**,**i**,**k**) High-magnification image of the sample surface depicted in panels **d**,**f**,**h**,**j** (×5000).

Figure 7 shows representative SEM images of the enamel block surfaces from different specimen groups obtained after six days of incubation. Before incubation, the enamel blocks exhibited smooth and polished surfaces with visible polishing lines and without prism outlines (Figure 7a). However, in the case of Group 1 containing only enamel blocks, enamel rods appeared on the block surfaces due to demineralization (Figure 7b,c). Meanwhile, no morphological changes were observed for the enamel specimens incubated with FXU, FIXE, CDR, and BC disks (Group 2), and their surfaces were similar to the polished surfaces of the control specimens (Figure 7d–k).

## 4. Discussion

In the present study, we investigated the protective effects of GIC and S-PRG restorative materials on the enamel surface immersed in lactic acid. The oral environment was simulated by adjusting the pH values of the test solutions to 4.0 each day. We found that the solutions containing only enamel blocks increased their pH values to approximately 5.1 after 24 h. The rods of the enamel blocks were visible in the obtained SEM images, owing to the loss of mineral tissue caused by the exposure to a low-pH environment. Furthermore, a very strong correlation was observed between the rate of release of Ca ions and the degree of enamel demineralization. However, the pH values of the solutions containing discs of restorative materials increased rapidly from 4.0 to more than 4.9 within three hours; as a result, the demineralization of the enamel blocks did not occur. SEM observations confirmed the absence of morphological changes of these blocks even after their exposure to a low-pH environment for six days. The Al, B, F, Na, Si, Sr, and Zn ions released from the GIC and S-PRG discs neutralized the reaction solution quickly and inhibited enamel demineralization at the early stage of the incubation process. Therefore, the null hypothesis, stating that the GIC- and S-PRG-containing restorative materials would not inhibit the demineralization of enamel, was rejected based on the obtained results. The observed effects of these restoratives on pH neutralization indicated that such demineralization indeed occurred during the short-term exposure to low-pH conditions.

The Al, Ca, F, Na, Si, Sr, and Zn ions released from the GIC- and S-PRG-containing restorative materials inhibited the demineralization process through neutralization. The glasses present in GICs are composed of calcium fluoro-alumino-silicates; hence, Al and Si are their primary network-forming elements. When the glass and acid components of GICs are mixed, an acid–base setting reaction is initiated, owing to the cross-linking of the carboxylic acid groups present in the aqueous solution of polyacrylic acids with the Al and Ca ions released from the glass powder [20]. The GICs continue to harden over time, leading to the formation of silicate or phosphate networks [10]. Because the electronic structure of Sr is very similar to that Ca, several Ca-containing glasses have been replaced with Sr-containing ones in some GIC products to enhance their radiopacity [21]. In this study, the release of Ca was observed only for CDR, a recently developed Ca-containing product for remineralizing and strengthening the tooth substrate. Furthermore, a small amount of Ca ions was released in Group 3. However, except for CDR, the results obtained for Group 3 were unexpected because the FXU, FIXE, and BC disks did not contain Ca element. Thus, the Ca ions released from these materials likely originated from some contaminants present in the incubation media or traces remained after the ICP tests. In any case, their contents were negligibly low. Analyses of the elemental distributions on the surfaces of FX-II and Fuji IX cements conducted by energy-dispersive X-ray spectroscopy and X-ray photoelectron spectroscopy confirmed the presence of Ca in their glass cores. However, it was also found that when the cement matrix was immersed into a CaCl_2_ solution, it absorbed Ca ions from it. These ions formed chemical bonds with the carboxylic acid groups of the cement matrix to produce a polyacid salt matrix that increased surface hardness [22]. Moreover, P ions were also likely present in the reaction solution (their effects on the release of ions from the surfaces of glass particles as well as on the working and setting times of GICs have been studied previously [23]). In this work, the small amount of P ions released by the disc-like specimens in Group 2 was related to the dissolution of some components of the demineralized enamel. Furthermore, the P release profiles of Groups 2 and 3 were similar. However, the concentration of P ions released in Group 3 was very low as that group consisted of only discs of the investigated restoratives. In general, the differences in the amounts of ions released by various cements can be attributed to the differences in their chemical compositions.

Both F and Sr ions play important roles in enamel restoration. Fluoride ions promote the formation of hard tissue by precipitating a calcium-fluoride-like (fluoro-apatite) layer onto the tooth surface, thus reinforcing the tooth structure [24]. In addition, they enhance the remineralization of partially demineralized enamel during the early stages of caries formation in the presence of Ca and PO_4_^3–^ ions from the saliva. The fluoridated enamel is more acid-resistant than the native enamel [25]; hence, fluoride-releasing materials may serve as reservoirs that increase the fluoride levels in the saliva, plaque, and dental hard tissues. In addition, fluoride helps increase the longevity of restorations by increasing the size of the acid-resistant zone adjacent to the cavity wall, thereby preventing further losses of mineral ions [1,10]. Meanwhile, Sr ions reinforce the tooth structure by converting hydroxyl apatite into Sr apatite, which results in the formation of an acid-resistant layer on the tooth surface [26]. They also promote remineralization and antibacterial activity while exhibiting a synergistic effect in the presence of F ions [27,28].

The structure of S-PRG fillers with a stable glass ionomer phase allows the release of Al, B, F, Na, Si, and Sr ions in resinous sealants [29]. In a previous study on coating materials [16], we found that the concentration of Na ions in a test solution was the highest one followed by those of B, Si, Sr, F, and Al ions. The concentration of each ion reached maximum after 24 h and then decreased over time. Although the amounts of released ions decreased gradually, enamel demineralization was inhibited in this process. Thus, the release of even small ion concentrations can produce a strong inhibitory effect on the demineralization process. The above-listed ions can effectively neutralize acidic plaque and promote anticaries activity in clinical settings. Moreover, the anticariogenic activity of F ions increases when the latter are combined with Al, Na, Si, or Sr ions. For this reason, the S-PRG discs used in the present study functioned as buffers that made the test solutions less cariogenic by reducing their acidity during the incubation period.

The obtained experimental results confirmed that the GIC- and S-PRG-containing restorative materials increased the pH of the acidic environment to the neutral level and reduced the likelihood of tooth caries through the localized protection of the tooth surface from acid attacks. The values of ion release obtained in our study demonstrated a similar tendency to those demonstrated in previous studies on GICs [11,12,30] and S-PRG filler restoratives [16,18,29,31]. Note that B ions were detected only in the BC solutions, while Ca and Zn ions were released only in the CDR ones. The fluoride leaching from GICs produces an antibacterial effect and helps prevent the formation of secondary caries [8]. Furthermore, the antibacterial effect of the B and F ions released by S-PRG fillers in resin composites inhibits the metabolism of *Streptococcus mutans* and limits the acid production [31]. Thus, to prevent the formation of dental biofilms and caries, it is necessary to develop bioactive dental restorative materials with enhanced antibacterial characteristics.

However, we note that during this study, measurements were only collected over six days; hence, the fact that the clinical efficacy can be determined in such a short time must be addressed. Consequently, it may be necessary to measure and conduct in vitro experiments over a longer period of time (e.g., one month).

## 5. Conclusions

It was found that GIC- and S-PRG-containing restoratives could serve as buffers for lactic acid solutions that rapidly increased their pH values to the neutral level by releasing various ions, which inhibited enamel demineralization. The bioactive properties of these materials make them suitable for the clinical restoration of early carious lesions in a biomimetic, atraumatic, and non-cavitated manner.

## Figures and Tables

**Table 1:** Materials used in this study

Material/(Code)	Manufacturer	Composition/Filler	Lot No.
GlasIonomer FX ULTRA	Shofu Inc.	Powder: fluoroaluminosilicate glass, polyacrylic acid	P: 061049
/(FXU)		Liquid: polyacrylic acid, polybasic carboxylic acid,	L: 061034
		distilled water	
Fuji IX GP Extra	GC Corp.	Powder: fluoroaluminosilicate glass, pigments,	P: 1108171
/(FIXE)		fluorescent material	
		Liquid: acrylic acid-tricarboxylic acid co-polymer,	L: 1108171
		tartaric acid, distilled water	
CAREDYNE RESTORE	GC Corp.	Powder: fluoroaluminosilicate glass,	P: 1812121
/(CDR)		fluorozincsilicate glass	
		Liquid: acrylic acid-tricarboxylic acid co-polymer,	L: 1812071
		polyacrylic acid, distilled water	
PRG Barrier Coat	Shofu Inc.	Base: S-PRG glass fillers, distilled water	P: 031503
/(BC)		methacrylic acid monomer, others	
		Active: Phosphoric acid monomer,	L: 111711
		bis-MPEPP, carboxylic acid monomer, TEGDMA,	
		photoinitiator, methacrylic acid monomer,	
		polybasic carboxylic acid, others	

S-PRG: surface pre-reacted glass ionomer, bis-MPEPP: 2,2′-bis (4-methacryloxy polyethoxyphenyl) propane, TEGDMA: triethylene glycol dimethacrylate.
